# Acupuncture and related therapies used as add-on or alternative to prokinetics for functional dyspepsia: overview of systematic reviews and network meta-analysis

**DOI:** 10.1038/s41598-017-09856-0

**Published:** 2017-09-04

**Authors:** Robin S. T. Ho, Vincent C. H. Chung, Charlene H. L. Wong, Justin C. Y. Wu, Samuel Y. S. Wong, Irene X. Y. Wu

**Affiliations:** 1 Jockey Club School of Public Health and Primary Care, The Chinese University of Hong Kong, Shatin, Hong Kong; 2 Hong Kong Institute of Integrative Medicine, The Chinese University of Hong Kong, Shatin, Hong Kong; 3 Cochrane Hong Kong, The Chinese University of Hong Kong, Shatin, Hong Kong

## Abstract

Prokinetics for functional dyspepsia (FD) have relatively higher number needed to treat values. Acupuncture and related therapies could be used as add-on or alternative. An overview of systematic reviews (SRs) and network meta-analyses (NMA) were performed to evaluate the comparative effectiveness of different acupuncture and related therapies. We conducted a comprehensive literature search for SRs of randomized controlled trials (RCTs) in eight international and Chinese databases. Data from eligible RCTs were extracted for random effect pairwise meta-analyses. NMA was used to explore the most effective treatment among acupuncture and related therapies used alone or as add-on to prokinetics, compared to prokinetics alone. From five SRs, 22 RCTs assessing various acupuncture and related therapies were included. No serious adverse events were reported. Two pairwise meta-analyses showed manual acupuncture has marginally stronger effect in alleviating global FD symptoms, compared to domperidone or itopride. Results from NMA showed combination of manual acupuncture and clebopride has the highest probability in alleviating patient reported global FD symptom. Combination of manual acupuncture and clebopride has the highest probability of being the most effective treatment for FD symptoms. Patients who are contraindicated for prokinetics may use manual acupuncture or moxibustion as alternative. Future confirmatory comparative effectiveness trials should compare clebopride add-on manual acupuncture with domperidone add-on manual acupuncture and moxibustion.

## Introduction

### Rationale

Functional dyspepsia (FD) is defined as pain or discomfort of the upper digestive tract in the absence of an organic cause that readily explains them^1^. One or more of the following symptoms are usually observed: postprandial fullness, early satiation, epigastric pain or burning^2^. FD is classified into two subtypes, postprandial distress syndrome (PDS, characterized by postprandial fullness and early satiation) and epigastric pain syndrome (EPS, characterized by epigastric pain and epigastric burning)^2^. The prevalence of FD ranged from 12 to 15% in the general population^3^. FD significantly reduces quality of life of patients, hence contributes to significant disease burden, treatment cost and loss of productivity^4^.

Current guidelines and expert consensus^5–9^ recommend the use of prokinetics as one of the routine treatments for FD. Effectiveness of prokinetics is however unsatisfactory, with a number needed to treat (NNT) of 16^10^. In addition, potential side effects of prokinetics have raised concern on their longer term use. For instance, existing studies suggest association between prokinetics use and increased risk of extra-pyramidal reactions, cardiac arrhythmic side effects including sudden cardiac death and drug-induced neurological disorders^11–14^. There is a need for addressing the effectiveness gap of those who are experiencing limited benefits from prokinetics, or those who are contraindicated to them.

In traditional Chinese medicine, acupuncture and related therapies have been used for treating functional gastrointestinal disorders (FGIDs) including FD^15, 16^. Existing evidence has shown the efficacy of acupuncture beyond sham control. In a Cochrane review of three trials, meta-analyses indicated that manual acupuncture was superior to sham acupuncture, for improving quality of life measured by SF-36 and Nepean Dyspepsia Life Quality Index^17^. Another meta-analysis performed by Kim and colleagues has shown that manual acupuncture was superior to sham acupuncture in FD symptoms reduction^18^. In a meta-analysis performed by Zhou and colleagues^19^, they have also shown that both manual acupuncture and electroacupuncture were superior to sham acupuncture, in the improvement of Nepean Dyspepsia Index.

When acupuncture and related therapies were compared with prokinetics, evidence from existing systematic reviews (SRs) is inconsistent. One SR showed similar effectiveness in FD symptoms reduction between manual acupuncture or electroacupuncture versus domperidone, as well as electroacupuncture versus itopride, in three separate trials^17^. However, meta-analyses from three other SRs showed that acupuncture and related therapies were more effective than prokinetics for FD symptoms reduction^18–20^.

These heterogeneous results make it difficult to draw conclusions on the effectiveness of acupuncture and related therapies on FD, used as an add-on or alternative to prokinetics. There is a need to perform an overview of SRs to clarify such uncertainty, as well as to assess the comparative effectiveness among different types of acupuncture and related therapies.

### Objectives

We conducted an overview of SRs to critically appraise and synthesize all clinical evidence on the comparative effectiveness of different acupuncture and related therapies on the treatment of FD, using a network meta-analysis (NMA) approach^21^.

## Methods

### Search methods for identification of studies

Four electronic international (Cochrane Database of Systematic Reviews, Database of Abstracts of Reviews of Effect, MEDLINE, and EMBASE) and four Chinese electronic databases (Wan Fang Digital Journals, China National Knowledge Infrastructure, Taiwan Periodical literature databases and Chinese Biomedical Database) were searched for potential SRs from their inception till November 2015. Validated, sensitivity maximized search filters for systematic reviews were applied in MEDLINE and EMBASE searches^22, 23^. The searches were limited to human studies and no language restriction was applied. The search strategies are presented in Appendix 1.

### Types of studies

To be included in this overview, SRs must include meta-analysis results, and satisfy the participants, interventions, controls and outcomes of interest criteria described below. SRs which only reported data narratively were excluded.

### Types of participants

Patients diagnosed with FD according to Rome criteria, or other criteria stated by the authors were considered. There was no restriction on the versions of Rome criteria used.

### Types of intervention

In this overview of SR, we only include three specific modalities: manual acupuncture, electroacupuncture, and moxibustion, as defined in Table 1 ^24^. Accordingly, in this overview of SR we defined “acupuncture and related therapies” as single or combined use of manual acupuncture, moxibustion and electroacupuncture.

**Table 1:** Definitions of modalities of acupuncture and related therapies in this overview of systematic review.

Manual acupuncture	Needle insertion into acupuncture points, followed by manual manipulation. The function of needling is believed to be to promote Qi (the vital energy) in the meridians in order to produce its therapeutic effect.
Moxibustion	A method in which a moxa herb is burned above the skin or on the acupuncture points. It can be used as a cone stick, loose herb, or applied at the end of the acupuncture needles. The purpose of moxibustion is to apply heat to the acupuncture points to alleviate symptoms.
Electroacupuncture	One type of modern acupuncture technique used with manual acupuncture, where needle is attached to a trace pulse current after it is inserted to the selected acupoint for the purpose of producing synthetic effect of electric and needling stimulation.

Accordingly, acupuncture and related therapies including the single or combined use of manual acupuncture, moxibustion, electroacupuncture were considered eligible for this overview. Prokinetics can be used as an add-on or alternative to these interventions. Prokinetics which are available in the market were eligible in the comparison group except cisapride. We chose to exclude cisapride as it has been removed from market due to serious adverse events^25^. Trials which evaluate combined therapy of proton pump inhibitors (PPIs) and prokinetics was excluded, as substantial side effects of their combined use have been shown in recent meta-analyses^26^. Combined therapy of H_2_ histamine receptor antagonist (H2RA) and prokinetics was also excluded, as H2RA has shown to be associated with an increased risk of pneumonia by a meta-analysis^27^, vitamin B12 deficiency by a case-control study^28^ and impaired cognitive function among elderlies by a cohort study^29^.

### Outcome measures

Trials results reported from each meta-analysis should include at least one of the following outcomes:

(i) Alleviation of dyspeptic symptoms, measured with either global or individual dyspepsia symptom scores; or (ii) proportion of patients achieving satisfactory alleviation of global or individual symptoms. Choices of these outcomes were based on current expert recommendations on endpoints for FD clinical trials^30^.

### Eligibility Assessment and Data Extraction

Two reviewers (RH and IW) independently screened titles and abstracts of retrieved citations, evaluated potential full texts, and determined eligibility. For each eligible SRs, full texts of each embedded RCTs were obtained. For duplicate citations, the most updated RCTs were selected for data extraction, while the older versions were used as supplementary information, if necessary. Discrepancies were resolved by consensus between two reviewers. A third reviewer (VC) was invited for consensus adjudication if discrepancy were not resolved.

The following were extracted from each embedded RCTs: year of publication, number of patients enrolled, participant characteristics, duration of FD diagnosis, diagnostic criteria used, and features of interventions in treatment and control groups. These features included frequencies of acupuncture sessions and prokinetics dosage intake. Types of outcome assessment, treatment duration, and follow-up duration, as well as any reported adverse events were also extracted.

### Methodological quality of included SRs and risk of bias assessment of included RCTs

The validated Methodological Quality of Systematic Reviews (AMSTAR) instrument was used to appraise quality of included SRs^31^. For each embedded RCTs, their risks of bias were assessed by the Cochrane’s risk of bias tool^32^. Both appraisals were performed by two reviewers (RH and IW) independently. Discrepancies were resolved by discussion between two reviewers, and consensus adjudication was sought from a third author (VC) if discrepancy persisted.

### Data synthesis

We followed established methods of conducting pairwise meta-analysis, followed by network meta-analysis (NMA) in this systematic review, which are considered as standard modelling methodology in the field^33^.

The first method, pairwise meta-analysis, synthesize results from head to head comparison between acupuncture and related therapies versus prokinetics under random effect model^34^.

Random effect pairwise meta-analyses were used to synthesize data extracted from embedded RCTs, separately for each type of acupuncture and related therapies using Review Manager Version 5.3^35^. Pooled relative risk (pooled RR) and standardized mean difference (SMD), with their 95% confidence interval (CI) were used to synthesize dichotomous outcome and continuous outcome respectively. I-square (I^2^) values were calculated for quantifying heterogeneity among RCTs. The I^2^ value of <25%, 26-50%, >50% were regarded as low, moderate, and high heterogeneity respectively^36^.

The outcome of global assessment on a Likert scale measures overall symptom improvement or deterioration. This approach allows the individual to integrate all aspects of one’s condition into a single treatment outcome, and is particularly suitable to show deterioration^30^. All primary outcomes of symptoms improvement were reported on short, 3 or 4 points Likert scales. Following recommendation of the Cochrane Handbook, we lumped these “short” ordinal results into dichotomous data as this resemble clinical decision making process of “to do” or “not to do”^37^. Provided that the ordinal outcome rating scales used among trials were similar, such combination of short ordinal scale is justified^38^. Indeed, binary assessment for overall symptom improvement is an accepted approach for outcome measurement in functional dyspepsia trials^39^.

Accordingly all 3 point Likert scale results were categorized as “marked improvement”, “slight improvement” and “no improvement”, while all 4 point Likert scale cases were categorized as “symptom-free”, “marked improvement”, “slight improvement” and “no improvement”. Then, in the transformation process, the categories of “symptom-free”, “marked improvement” or “slight improvement” cases were combined and labelled as “favourable” cases; while “no improvement” cases were renamed as “unfavourable” cases.

The second method is indirect comparisons of the effectiveness among 11 treatments of acupuncture and related therapies used as add-on or alternative to prokinetics for FD via NMA, a standard modelling methodology for conducting overview of systematic review^40^. NMA is a preferred approach which offers a set of methods to visualize and interpret wider picture of existing evidence, as well as to understand the comparative effectiveness of these multiple treatments^41^.

NMA was conducted to explore the highest probability of being the most effective form of acupuncture and related therapies when compared to prokinetics, either alone or as an add-on, by using STATA Version 13.0 (STATA Corporation, College Station, TX)^42^. Indirect comparisons of dichotomous and continuous outcomes among different treatments were implemented with the mvmeta command^43, 44^. Assumption of NMA was checked by evaluating inconsistency factor (IF) of direct and various indirect effect estimates, using the loop-specific heterogeneity estimates^43^ for the same comparison.

When summarizing comparative effectiveness ranking results from NMA, we calculated the probability of an intervention being the most effective treatment, the second best treatment, the third best treatment and so on by calculating the RR (dichotomous outcome) and mean difference (continuous outcome) for each possible pair of comparisons. The probability for a treatment of being at a particular rank was interpreted by the surface under the cumulative ranking curve (SUCRA) and mean ranks were used to obtain the effectiveness hierarchy. The more surface of SUCRA curve, the higher probability of the treatment will be^43, 44^. All statistical tests were 2-tailed with significance level of 0.05.

## Results

### Literature search

**Figure 1:**
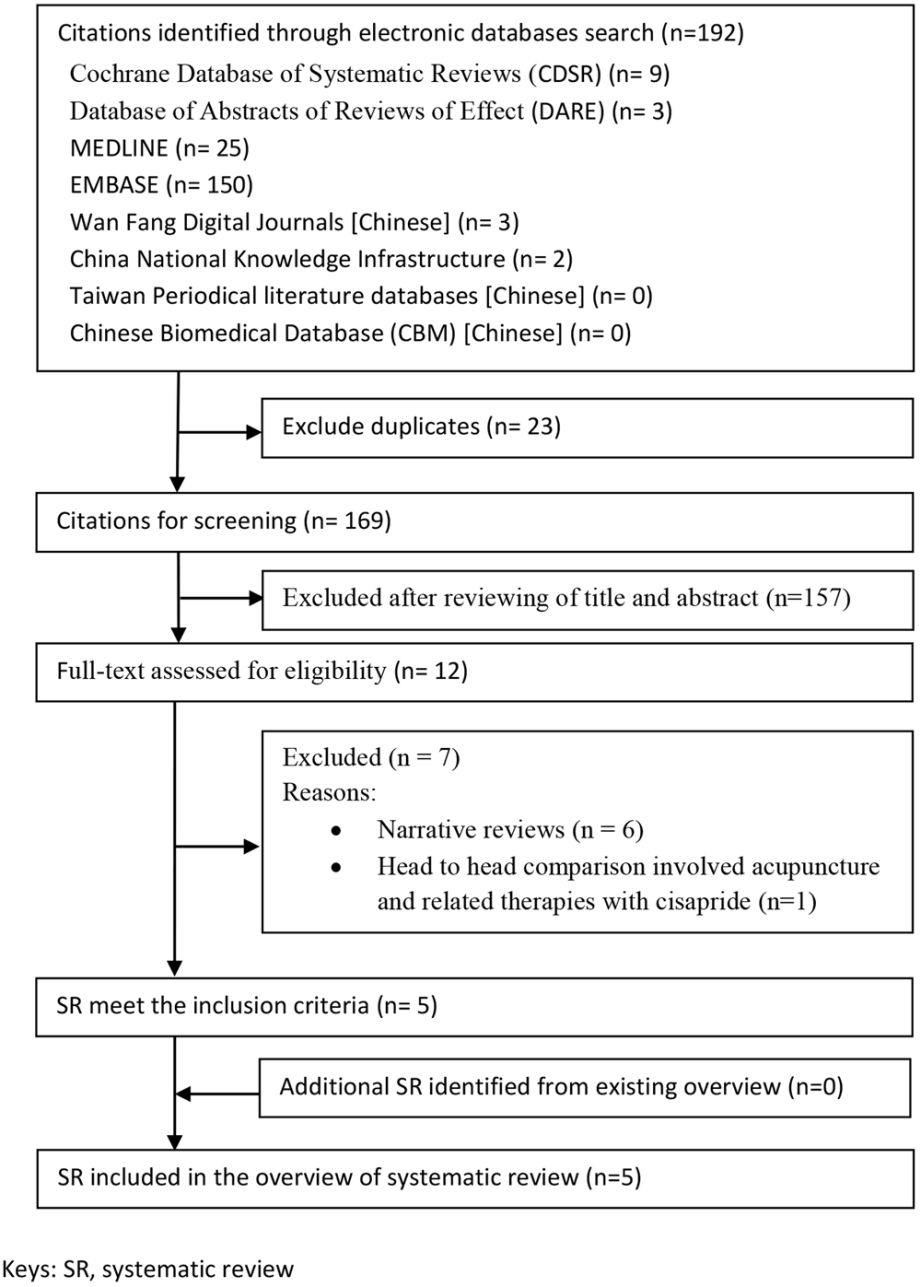
Flowchart of literature selection on systematic reviews on acupuncture and related therapies for functional dyspepsia.

The search strategies yielded 192 records, and 23 duplicates were identified and excluded. We excluded 157 citations after screening titles and abstracts, and full texts of the remaining 12 citations were retrieved for further assessment. Seven publications were excluded for the following reasons: six were narrative reviews, and one SR involved head to head comparison of acupuncture and related therapies with cisapride. A total of 5 SRs were included in this overview. Details of the literature search and SR selection can be found in Fig. 1. These 5 SRs (Appendix 2) included a total of 78 RCTs. Among these RCTs, fifty-six were excluded due to the following reasons:

- duplicate publications (n = 24);
- ineligible control groups (n = 29), of which 4 groups were PPIs plus prokinetics, 3 groups were Chinese herbal medicine, 1 group was homeopathy, 1 group was antacid, 1 group was H_2_ histamine receptor antagonist plus prokinetics, 7 groups were head to head comparison between acupuncture and related therapies with cisapride and 12 used sham acupuncture;
- ineligible experimental groups (n = 2) of vitamin injection and finger acupressure;
- one did not report outcome on FD symptom changes (n = 1).

**Figure 2:**
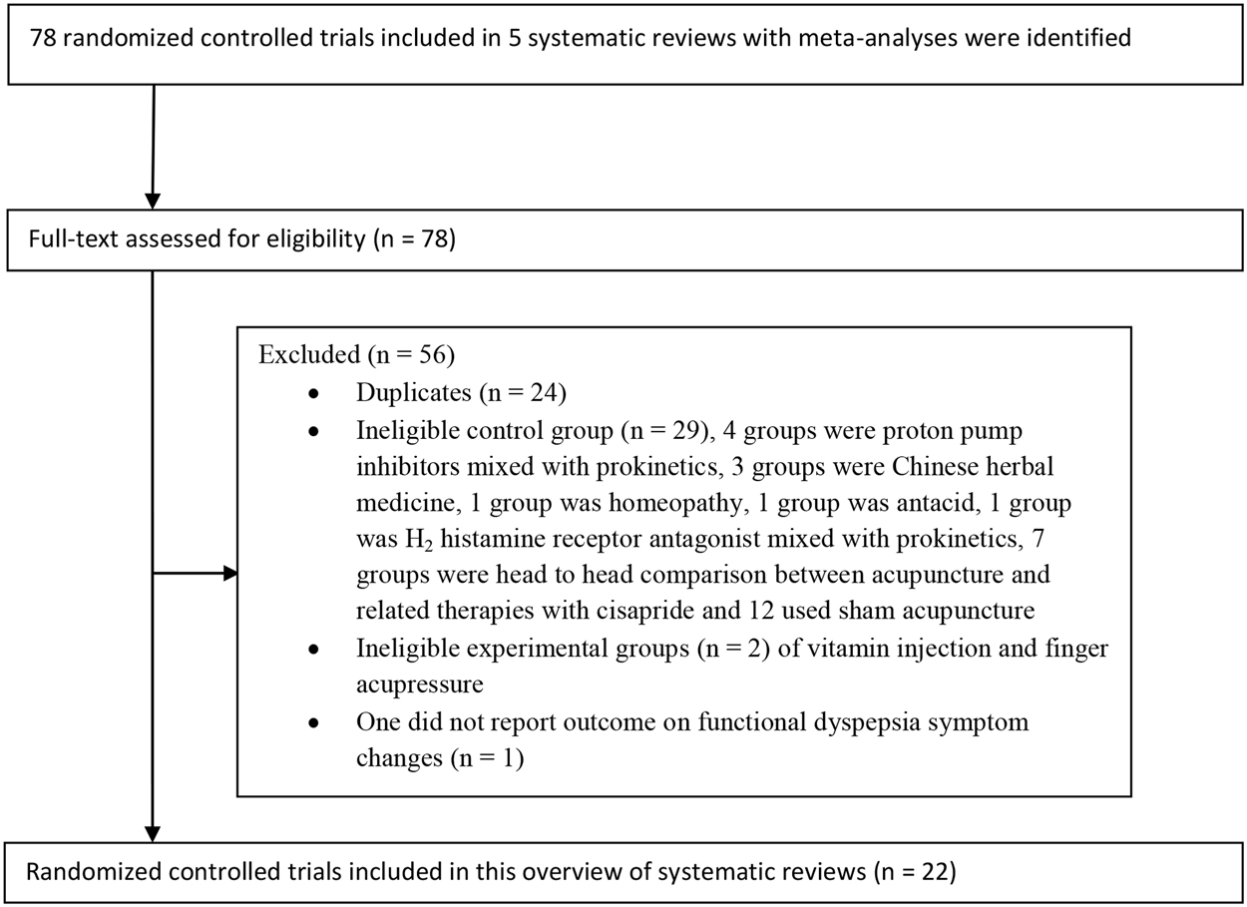
Flowchart for randomized controlled trials selection for acupuncture and related therapies for functional dyspepsia.

After applying eligibility criteria, 22 unique RCTs were extracted from the SRs for inclusion in this overview. Details on the RCTs literature selection were presented in Fig. 2. A list of these eligible RCTs was presented in Appendix 3.

### Characteristics of included RCTs

#### Participants

Characteristics of included RCTs were summarized in Table 2. The 22 RCTs included a total of 1,727 FD patients. Age of participants ranged from 17 to 70 years. Average sample size of the RCTs was 79 participants (ranging from 46 to 260). Duration of diagnosis ranged from 1.2 to 120 months.

**Table 2:** Main characteristics of included randomized controlled trials.

First author, year of publication (Country)	No. of participants R/A*	Age: mean $\pm$ SD or range (years)	Duration of FD diagnosis: Range or mean $\pm$ SD (months)	Diagnostic criteria	Intervention	Control	Time of follow- up for FD symptoms assessment	Adverse events reported	Type of outcomes
Tang 2006 (China)	I: 32/32 C: 30/30	I: 38.2 $\pm$ 11.3 C: 36.7 $\pm$ 12.8	Range: I: 3-120 C: 3-108	Rome II	Manual Acupuncture	Domperidone	4 weeks	NR	Patient reported FD symptom score on a 4 point Likert scale (symptom-free, marked improvement, slight improvement, and no improvement)
Liu 2001 (China)	I: 38/38 C: 30/30	I: 43.6 ± 14.7 C: 42.9 ± 14.6	Mean I: 38 C: 39	Rome I	Manual acupuncture	Domperidone	4 weeks	NR	Patient reported FD symptom score on a 4 point Likert scale (symptom-free, marked improvement, slight improvement, and no improvement)
Xu 2005 (China)	I: 45/45 C: 42/42	I: 40.20 ± 4.12 C: 39.38 ± 4.52	Mean $\pm$ SD: I: 20 ± 9 C: 20 ± 8	Rome I	Manual acupuncture	Domperidone	4 weeks	NR	Patient reported FD symptom score on a 4 point Likert scale (symptom-free, marked improvement, slight improvement, and no improvement)
Feng 2004 (China)	I: 35/35 C: 30/30	I: 41.9 ± 12.8 C: 42.2 ± 11.5	Mean: I:41 C:38	Rome I	Manual acupuncture	Domperidone	2 weeks 4 weeks	NR	Patient reported FD symptom score on a 4 point Likert scale (symptom-free, marked improvement, slight improvement, and no improvement)
Wang 2002 (China)	I: 45/45 C: 36/36	I: 48 ± 22 C: 47 ± 21	Range: I: 1.5-120 C: 1.3-96	Diagnostic criteria were based on FD symptoms onset at least 5 weeks.	Manual acupuncture	Domperidone	2 weeks	NR	Patient reported FD symptom score on a 3 point Likert scale (marked improvement, slight improvement, and no improvement)
Wu 2010 (China)	I: 35/35 C: 35/35	Mean: I:47 C:47	NR	Rome III	Manual acupuncture	Domperidone	4 weeks	NR	Patient reported FD symptom score on a 3 point Likert scale (marked improvement, slight improvement, and no improvement)
Sun 2004 (China)	I: 26/26 C: 24/24	I: 43.55 $\pm$ 12.46 C: 42.41 $\pm 11.34$	Range: I:1.2-84 C:1.3-82	Rome II	Moxibustion	Domperidone	2 weeks	NR	Patient reported FD symptom score on a 4 point Likert scale (symptom-free, marked improvement, slight improvement, and no improvement)
Yang 2011 (China)	I: 23/23 C: 23/23	Range: I: 30-63 C: 28-64	Range: I: 36-48 C: 3-60	Rome III	Moxibustion	Domperidone	4 weeks	NR	Patient reported FD symptom score on a 4 point Likert scale (symptom-free, marked improvement, slight improvement, and no improvement)
Wang 2013 (China)	I: 40/40 C: 40/40	Range: I: 18-66 C: 21-64	Range: I: 5-72 C: 4-84	Rome II	Moxibustion	Domperidone	4 weeks	NR	Patient reported FD symptom score on a 4 point Likert scale (symptom-free, marked improvement, slight improvement, and no improvement)
Zhou 2005 (China)	I: 64/64 C: 62/62	NR	NR	Rome II	Electro-acupuncture	Domperidone	3 weeks	NR	Patient reported FD symptom score on a 3 point Likert scale (marked improvement, slight improvement, and no improvement)
Sun 2012 (China)	I: 50/50 C: 50/50	I: 37.33 C: 39.68	$NR$	Rome III	Manual acupuncture + moxibustion	Domperidone	4 weeks	NR	Patient reported FD symptom score on a 4 point Likert scale (symptom-free, marked improvement, slight improvement, and no improvement)
Zheng 2013 (China)	I: 30/30 C: 30/30	I: 34.77 $\pm$ 10.25 C: 34.03 $\pm$ 8.97	Mean $\pm$ SD: ${I:}18.50\pm 8.92$ C: 21.13 $\pm 9.98$	Rome III	Manual acupuncture + moxibustion	Domperidone	4 weeks	I: 4 cases reported ecchymosis at acupuncture points in intervention group C: 1 case rash and 2 cases of constipation in control group	Patient reported FD symptom score on a 4 point Likert scale (symptom-free, marked improvement, slight improvement, and no improvement)
Zhou 2013 (China)	I: 54/54 C: 54/54	Range: I: 21-63 C: 23-65	Range: I: 5-60 C: 8-60	Diagnostic criteria were based on FD symptoms onset at least 3 months.	Manual acupuncture + moxibustion added on domperidone	Domperidone	4 weeks	Adverse events NR in detailed	Patient reported FD symptom score on a 4 point Likert scale (symptom-free, marked improvement, slight improvement, and no improvement)
Hu 2012 (China)	I: 34/34 C: 36/36	I: 45.21 ± 9.37 C: 44.81 ± 8.95	Mean $\pm$ SD: I: 23.68 ± 14.66 C: 23.89 ± 13.13	Rome III	Manual acupuncture	Itopride	2 weeks 8 weeks	NR	Patient reported FD symptom score on a 4 point Likert scale (symptom-free, marked improvement, slight improvement, and no improvement)
Jin 2013 (China)	I: 36/34 C: 36/36	I: 45.21 ± 9.37 C: 44.81 ± 8.95	Mean $\pm$ SD: I: 23.68 ± 14.66 C: 23.89 ± 13.13	Rome III	Manual acupuncture	Itopride	2 weeks 8 weeks	NR	Patient reported FD symptom score on a 4 point Likert scale (symptom-free, marked improvement, slight improvement, and no improvement)
Chen 2013 (China)	I: 30/30 C: 30/30	I: 45.3 ± 11.8 C: 46.2 ± 12.3	Mean $\pm$ SD: I: 20.5 ± 7.8 C: 20.6 ± 7.6	Rome III	Manual acupuncture	Itopride	4 weeks	NR	Patient reported FD symptom score on a 4 point Likert scale (symptom-free, marked improvement, slight improvement, and no improvement)
Zhang 2009 (China)	I: 24/24 C: 24/24	I: 35.7 ± 10.43 C: 35.23 ± 11.25	Mean $\pm$ SD: I: 18.4 ± 12.72 C: 18.17 ± 13.54	Rome III	Electro-acupuncture	Itopride	4 weeks	NR	Patient reported FD symptom score on a 4 point Likert scale (symptom-free, marked improvement, slight improvement, and no improvement)
Yang 2009 (China)	I: 40/40 C: 40/40	I: 46.2 ± 11.7 C: 45.9 ± 12.1	Mean $\pm$ SD: I: 14.5 ± 7.8 C: 14.7 ± 7.6	Rome III	Electro-acupuncture	Itopride	4 weeks	NR	Patient reported FD symptom score on a 4 point Likert scale (symptom-free, marked improvement, slight improvement, and no improvement)
Shi 2011 (China)	I: 42/42 C:42/42	I: 43.63 ± 10.78 C: 42.38 ± 11.19	Mean $\pm$ SD: I: 19.21 ± 20.85 C: 21.15 ± 18.91	Rome III	Manual acupuncture + moxibustion	Itopride	4 weeks	NR	Patient reported FD symptom score on a 4 point Likert scale (symptom-free, marked improvement, slight improvement, and no improvement)
Xu 2014 (China)	I: 21/21 C:21/21	Range: I: 17-68 C: 20-69	Range: I: 7-60 C: 5-72	Rome II	Manual acupuncture + moxibustion	Mosapride	4 weeks	NR	Patient reported FD symptom score on a 3 point Likert scale (marked improvement, slight improvement, and no improvement)
He 2012 (China)	I: 130/130 C: 130/130	Range: I: 29-67 C: 28-66	Range: I: 12-60 C: 12-56	Rome II	Manual acupuncture added on mosapride	Mosapride	4 weeks 12 weeks	I: 6 minor nausea, 5 increase defecation & 5 stomach rumble C: 5 minor nausea, 4 increase defecation & 5 stomach rumble	Patient reported FD symptom score on a 3 point Likert scale(marked improvement, slight improvement, and no improvement)
Liu 2011 (China)	Group 1 Manual acupuncture + clebopride: 40/40	Combined group: 48.3 $\pm$ 4.8	Combined group: 6.8 $\pm$ 1.1	Rome III	Group 1: Manual acupuncture	Group 3: Clebopride	4 weeks	NR	Patient reported FD symptom score on a 3 point Likert scale (marked improvement, slight improvement, and no improvement)
	Group 2 Manual acupuncture: 38/38	Manual acupuncture group: 45.5 $\pm$ 5.7	Manual acupuncture group: 7.7 $\pm$ 0.3		added on clebopride				
	Group 3 Clebopride:38/38	Clebopride group: 46.0 $\pm$ 5.0	Clebopride group: 7.0 $\pm$ 0.5		Group 2:Manual acupuncture				

*R: Number of patients randomized, A: Number of patients analyzed; ^#^ I: Intervention group, C: Control group; FD, functional dyspepsia; SD, standard deviation; NR, Not reported.

#### Diagnostic criteria

Respectively eleven, six and three RCTs applied the Rome III, Rome II and Rome I criteria. Two RCTs followed criteria determined by the authors.

#### Interventions

Amongst the 22 included RCTs, all RCTs were two arm trials, except one which has three arms. Twenty-three comparisons were therefore included in this review. Four different forms of acupuncture and related therapies were evaluated: manual acupuncture (10 comparisons), manual acupuncture plus moxibustion (4 comparisons), moxibustion (3 comparisons), electroacupuncture (3 comparisons). Three types of combination therapies were evaluated: one each for clebopride or mosapride being an add-on to manual acupuncture (2 comparisons) and domperidone being an add-on to manual acupuncture plus moxibustion (1 comparison). Four types of oral prokinetics were evaluated as comparison: domperidone (13 comparisons), itopride (6 comparisons), mosapride (2 comparisons), and clebopride (1 comparison). Fifteen out of 22 RCTs offered 20 to 30 sessions of acupuncture and related therapies. Detailed descriptions of the included acupuncture and related therapies procedures are reported in Table 3 according to the revised standards for reporting interventions in clinical trials of acupuncture (STRICTA)^45^. Information including the style of acupuncture, names of acupuncture points used, depth of needle insertion or moxa distance away from skin, response sought, retention time, needle or moxa type, length and diameter of needle or moxa, frequency and duration of acupuncture sessions were shown in detailed in Table 3.

**Table 3:** Descriptions of the included acupuncture and related therapies.

First author, year of publication (Country)	Style of acupuncture	Names of acupuncture points used	Depth of needle insertion (Moxa distance away from skin)	Response sought	Retention time	Needle type, length & diameter (Moxa type, length & diameter)	Frequency & duration of acupuncture sessions	Type of prokinetics compared	Dosage and duration of prokinetics patients received	Practitioner background
Tang 2006 (China)	Manual Acupuncture	Bilateral Zusanli (ST 36) Neiting (ST44) Taichong (LR3) Neiguan (PC 6) Pishu (BL20) Weishu (BL21) Xinshu (BL15) Zhongwan (CV12)	NR	De-qi response	30 minutes	Needle type: No. 28-30 Length: 25mm Diameter: NR	1 session daily with a total of 30 sessions for 30 days/ 4 weeks	Domperidone	10 mg daily for 30 days/ 4 weeks	NR
Liu 2001 (China)	Manual acupuncture	Zhongwan (CV12) Zusanli (ST 36) Neiguan (PC 6) Hegu (LI4) Weishu (BL21) Pishu (BL20) Taichong (LR3) Qihai (CV6) Guanyuan (CV4) Tianshu (ST25)	NR	De-qi response	30 minutes	Needle type: No. 28 Length: NR Diameter: NR	1 session daily with a total of 30 sessions for 30 days/4 weeks, 2 days rest in-between 10 sessions	Domperidone	10 mg daily for 30 days/4 weeks	NR
Xu 2005 (China)	Manual acupuncture	Pishu (BL20) Weishu (BL21) Zhongwan (CV12) Tianshu(ST25) Qihai (CV6) Neiguan (PC 6) Gongsun (SP4) Zusanli (ST 36)	NR	Response from the feeling of needles	30 minutes	Needle type: No. 28 Length: NR Diameter: NR	1 session daily with a total of 30 sessions for 30 days/4 weeks, 2 days rest in-between 5 sessions	Domperidone	10 mg daily for 30 days/4 weeks	NR
Feng 2004 (China)	Manual acupuncture	Zusanli (ST 36) Zhongwan (CV12) Neiguan (PC 6) Taichong (LR3)	38mm	Responses of the sensation of numbness and soreness	30 minutes	NR	1 session daily with a total of 28 sessions for 4 weeks	Domperidone	10 mg daily for 4 weeks	NR
Wang 2002 (China)	Manual acupuncture	Zhongwan (CV12) Additional acupuncture points for patients who diagnosed with the syndrome differentiation of Liver Qi Invading the Stomach: Taichong (LR3) Coldness at Spleen and Stomach: Zusanli (ST 36)	76-127mm	De-qi response	20 minutes	Needle type: NRLength: 152mm Diameter: 0.4mm	1 session daily with a total of 10 sessions for 2 weeks, 2 days rest in-between 5 sessions	Domperidone	10 mg daily for 2 weeks	NR
Wu 2010 (China)	Manual acupuncture	Taichong (LR3) Neiguan (PC 6) Ganshu (BL18) Zhongwan (CV12) Zusanli (ST 36) Weishu (BL21) Baihui (GV20) Si Shen Cong (EX-HN-1) Shenmen (HT7)	NR	Responses of the sensation of numbness and soreness	30 minutes	Needle type: No. 30 Length: 38- 64mm Diameter: NR	1 session daily with a total of 24 sessions for 4 weeks, 2 days rest in-between 8 sessions	Domperidone	10 mg daily for 30 days/4 weeks	NR
Sun 2004 (China)	Moxibustion	Zhongwan (CV 12), Qihai (CV 6), Neiguan (PC 6), Gongsun (SP 4)	(NR)	Responses of flushing of the skin on acupuncture points	NR	(Moxa type: Ignited moxa pen Length: NR Diameter: NR)	1 session daily with a total of 10 sessions for 10 days/ 2 weeks	Domperidone	10 mg daily for 10 days/ 2 weeks	NR
Yang 2011 (China)	Moxibustion	Ganshu (BL18), Weishu (BL21), & acupuncture points between Shangwan (CV13) and Xiawan (CV10)	(30mm)	Responses of warmth on acupuncture points	Few minutes to 1 hour, it varies from patient to patient	(Moxa type: Ignited moxa stick Length: 200-210mm Diameter: 170-180mm)	1 session daily with a total of 16 sessions for 4 weeks, 1 day rest in-between each session	Domperidone	10 mg daily for 30 days/4 weeks	NR
Wang 2013 (China)	Moxibustion	Unilateral Shenque (CV8) Zhongwan (CV12) Guanyuan (CV4) Bilateral Tianshu(ST25) Liangmen (ST21) Shuidao (ST28) Neiguan (PC 6) Gongsun (SP4) Hegu (LI4) Taichong (LR3) Shousanli (LI10) Zusanli (ST 36)	(NR)	NR	NR	(Moxa type: Ignited moxa stick Length: NR Diameter: NR)	1 session daily with a total of 30 sessions for 30 days/4 weeks, 2 days rest in-between 10 sessions	Domperidone	10 mg daily for 30 days/4 weeks	NR
Zhou 2005 (China)	Electro-acupuncture	Unilateral Zhongwan (CV 12), Zusanli (ST 36), Sanyinjiao (SP 6), Hegu (LI 4) Bilateral Neiguan (PC 6) with electric current applying to Zusanli (ST 36) Sanyinjiao (SP 6)	NR	Once de-qi response has felt by patients, electric current were connected to needles.	20 minutes	NR	1 session daily with a total of 21 sessions for 3 weeks	Domperidone	10 mg daily for 3 weeks	NR
Sun 2012 (China)	Manual acupuncture + moxibustion	Zhongwan (CV12) Tianshu(ST25) Zusanli (ST 36) Additional acupuncture points for patients who diagnosed with the syndrome differentiation of Discordance between Liver and Stomach Qi: Taichong (LR3) Pishu (BL20) Ganshu (BL18) Deficiency of Spleen and Stomach: Pishu (BL20) Weishu (BL21) Dampness and Heat at the Spleen and Stomach: Xiawan (CV10) Neiting (ST44)	NR	Once de-qi response has felt by patients, moxa was ignited and applied on top of acupuncture needles.	30 minutes	Needle type: NR Length: NR Diameter: NR (Moxa type: NR Length: NRDiameter: NR)	1 session daily with a total of 28 sessions for 4 weeks.	Domperidone	10 mg daily for 4 weeks	NR
Zheng 2013 (China)	Manual acupuncture + moxibustion	Zhongwan (CV12) Zusanli (ST 36) Neiguan (PC 6) Linggu Moxa applied on Zhongwan (CV12) and bilateral Zusanli (ST 36)	(30-40mm)	Once de-qi response has felt by patients, moxa was ignited and applied on top of acupuncture needles.	30 minutes	Needle type: NR Length: 40mm Diameter: 0.25mm (Moxa type: Ignited moxa stick Length: 20mm Diameter: NR)	1 session daily with a total of 30 sessions for 30 days/4 weeks	Domperidone	10 mg daily for 30 days/4 weeks	NR
Zhou 2013 (China)	Manual acupuncture + moxibustion	Zhongwan (CV12) Zusanli (ST 36) Qihai (CV6) Neiguan (PC 6) Yinlingquan (SP9) Gongsun (SP4)	NR	De-qi response	NR	NR	1 session daily with a total of 16 sessions for 4 weeks, 1 day rest in-between each session	Domperidone	10 mg daily for 4 weeks	NR
Hu 2012 (China)	Manual acupuncture	Unilateral: CV12 (Zhongwan) Bilateral: ST36 (Zusanli), PC6 (Neiguan), ST25 (Tianshu) A dditional acupuncture points for patients who diagnosed with the syndrome differentiation of Liver Qi Stagnation: Danzhong (CV17) Zhangmen (LR13) Qi Deficiencies of the Spleen and Stomach: Pishu (BL20) Weishu (BL21) Liver Qi Invading the Stomach: Qimen (LR14) Taichong (LR3)Dampness and Heat at the Stomach: Neiting (ST44) Yinlingquan (SP9)	30-50mm	NR	30 minutes	Needle type: No. 25 Length: 40-50mm Diameter: 25mm	1 session daily with a total of 12 sessions for 2 weeks, 1 day rest in-between 6 sessions.	Itopride	50 mg daily for 2 weeks with one day rest in-between 6 days	NR
Jin 2013 (China)	Manual acupuncture	Unilateral: CV12 (Zhongwan) Bilateral: ST36 (Zusanli), PC6 (Neiguan), ST25 (Tianshu) Additional acupuncture points for patients who diagnosed with the syndrome differentiation of Liver Qi Stagnation: Danzhong (CV17) Zhangmen (LR13) Qi Deficiencies of the Spleen and Stomach: Pishu (BL20) Weishu (BL21) Liver Qi Invading the Stomach: Qimen (LR14) Taichong (LR3) Dampness and Heat at the Stomach: Neiting (ST44) Yinlingquan (SP9)	30-50mm	NR	30 minutes	NR	1 session daily with a total of 12 sessions for 2 weeks, 1 day rest in-between 6 sessions.	Itopride	50 mg daily for 12 days/ 2 weeks	NR
Chen 2013 (China)	Manual acupuncture	Unilateral: Zhongwan (CV12) Danzhong (CV17) Bilateral: ST36 (Zusanli), PC6 (Neiguan), ST25 (Tianshu)	NR	De-qi response	30 minutes	Needle type: NR Length: 40mm Diameter: 0.3mm	1 session daily with a total of 20 sessions for 4 weeks, 2 days rest in-between 5 sessions.	Itopride	50 mg daily for 4 weeks, with 2 days rest in-between 5 days.	NR
Zhang 2009 (China)	Electro-acupuncture	Unilateral Zusanli (ST 36) Bilateral: Zhongwan (CV12) Weishu (BL21)	NR	Once de-qi response has felt by patients, electric current were connected to needles.	30 minutes	Needle type: NR Length: 40mm Diameter: 0.25mm	1 session daily with a total of 20 sessions for 4 weeks, 2 days rest in-between 5 sessions.	Itopride	50 mg daily for 4 weeks, with 2 days rest in-between 5 days.	NR
Yang 2009 (China)	Electro-acupuncture	Chongyang (ST42) Fenglong (ST40) Zusanli (ST 36) Liangqiu (ST34) with electric current applying to Zusanli (ST 36) Liangqiu (ST34)	30-50mm	Once de-qi response has felt by patients, electric current were connected to needles.	30 minutes	Needle type: No. 30 Length: 25-50mm Diameter: 0.3mm	1 session daily with a total of 20 sessions for 4 weeks, 2 days rest in-between 5 sessions.	Itopride	50 mg daily for 4 weeks, with 2 days rest in-between 5 days.	NR
Shi 2011 (China)	Manual acupuncture+ moxibustion	Zhongwan (CV12) Neiguan (PC 6) Zusanli (ST 36) Tianshu(ST25) Moxa applied on Tianshu(ST25) Zhongwan (CV12) Guanyuan (CV4) Ganshu (BL18) Geshu (BL17) Shangjuju (ST37)	NR	Responses of warmth on the skin of stomach, with spreading to chest and back from moxibustion.	30 minutes	Needle type: No. 30 Length: 25-50mm Diameter: 0.3mm (Moxa type: Ignited moxa stick Length: NR Diameter: NR)	1 session daily with a total of 28 sessions for 4 weeks.	Itopride	50 mg daily for 4 weeks	NR
Xu 2014 (China)	Manual acupuncture + moxibustion	Zhongwan (CV12) Neiguan (PC 6) Zusanli (ST 36) Sanyinjiao (SP6)	NR	Once de-qi response has felt by patients, moxa was ignited and applied on top of acupuncture needles.	NR	Needle type: No. 30 Length: 25-64mm Diameter: NR (Moxa type: Ignited moxa stick Length: 15-20mm Diameter: NR)	1 session daily with a total of 30 sessions for 30 days/4 weeks, 2 or 3 days rest in-between 10 sessions.	Mosapride	5 mg daily for 30 days/4 weeks	NR
He 2012 (China)	Manual acupuncture	Zusanli (ST 36) Neiting (ST44) Taichong (LR3) Neiguan (PC 6) Weishu (BL21) Ganshu (BL18) Xinshu (BL15) Zhongwan (CV12)	NR	De-qi response	15-30 minutes	NR	1 session daily with a total of 28 sessions for 4 weeks	Mosapride	5 mg daily for 4 weeks	NR
Liu 2011 (China)	Manual acupuncture	Bilateral Zusanli (ST 36) Neiguan (PC 6) Tianshu(ST25)	NR	De-qi response	20-30 minutes	NR	Total number of sessions NR. Patients receive sessions for 4 weeks.	Clebopride	0.68 mg daily for 4 weeks	NR

Key: NR: Not reported; mm: millimeter.

Treatment duration for acupuncture or related therapies as well as prokinetics ranged from four weeks (17 RCTs) to three weeks (1 RCT) and two weeks (4 RCTs). Follow-up duration for outcome assessment ranged from 2 weeks to 12 weeks.

### Critical appraisal of SRs and RCTs

Methodological quality of the five SRs, including one Cochrane SR and four non-Cochrane SRs, was mediocre. All included SRs performed duplicate study selection and data extraction, conducted comprehensive literature search, formulated conclusions appropriately regarding to scientific quality of the included studies, and provided characteristics and assessed the scientific quality of included studies. Four SRs used appropriate statistical methods for combining findings. However, none of the SRs explicitly stated conflicts of interests for both the SR and included studies. Except one Cochrane SR, the remaining four did not fulfill the following three AMSTAR criteria: providing a protocol of the SR, searching unpublished literature, providing lists of both included and excluded studies. Only two SRs assessed publication bias. Details on methodological quality of the five SRs are presented in Table 4.

**Table 4:** Methodological quality of included systematic reviews on acupuncture and related therapies for functional dyspepsia (FD).

First author and publication year	AMSTAR item										
	1	2	3	4	5	6	7	8	9	10	11
Kim, 2015	N	Y	Y	NR	N	Y	Y	Y	Y	N	N
Lan, 2014	Y	Y	Y	Y	Y	Y	Y	Y	Y	N	N
Wu, 2015	N	Y	Y	NR	N	Y	Y	Y	N	Y	N
Zhu, 2008	N	Y	Y	NR	N	Y	Y	Y	Y	N	N
Zhou, 2016	N	Y	Y	NR	N	Y	Y	Y	Y	Y	N
# of Yes (%)	1(20.0)	5(100.0)	5(100.0)	1(20.0)	1(20.0)	5(100.0)	5(100.0)	5(100.0)	4(80.0)	2(40.0)	0 (0.0)

Keys: N, no; NR: not reported; N/A: Not applicable; Y, Yes (SR fulfilling the criteria); # of Yes: number of yes; AMSTAR: Assessing the Methodological Quality of Systematic Reviews. AMSTAR item: 1. Was an ‘a priori’ design provided? 2. Was there duplicate study selection and data extraction? 3. Was a comprehensive literature search performed? 4. Was the status of publication (i.e. grey literature) used as an inclusion criterion? 5. Was a list of studies (included and excluded) provided? 6. Were the characteristics of the included studies provided? 7. Was the scientific quality of the included studies assessed and documented? 8. Was the scientific quality of the included studies used appropriately in formulating conclusions? 9. Were the methods used to combine the findings of studies appropriate? 10. Was the likelihood of publication bias assessed? 11. Was the conflict of interest included?

Overall, risk of bias amongst included RCTs is moderate. Amongst these 22 RCTs, nine were of low risk of bias for random sequence generation, while seven did not report sequence generation procedures used and six were of high risk of bias. Except one RCT, all did not state details on allocation concealment. In all trials, patient blinding was not applied, and blinding of investigators was not reported. Outcome assessment was based on subjective outcome reported by patients themselves. Thus these 2 domains were subjected to high risk of bias. All included RCTs had low risk of bias for incomplete data, with 21 out of 22 RCTs achieved 100% follow up rate. Selective outcome reporting was unclear in all included RCTs as none of them provided published protocols. Risk of bias assessment results of the 22 RCTs were presented in Table 5.

**Table 5:** Risk of Bias among included randomized controlled trials.

Source (First author, year)	Random sequence generation	Allocation concealment	Blinding of participants and investigators	Blinding of outcome assessment	Incomplete outcome data addressed	Selective outcome reporting
Tang 2006	Low risk Random sequence was generated from a table of random numbers.	Unclear risk Authors did not state details.	High risk Use of blinding to patients was not applied and blinding of investigators was not reported.	High risk Outcome assessment was based on subjective outcome self-reported by patients themselves.	Low risk All participants completed the study. Drop-out rate: 0%	Unclear risk Protocol is not reported by authors.
Liu 2001	Low risk Random sequence was generated by computer.	Low risk Patients’ sequences were sealed in opaque envelopes.	High risk Use of blinding to patients was not applied and blinding of investigators was not reported.	High risk Outcome assessment was based on subjective outcome self-reported by patients themselves.	Low risk All participants completed the study. Drop-out rate: 0%	Unclear risk Protocol is not reported by authors.
Xu 2005	High risk Random sequence was generated according to the order of consultation.	Unclear risk Authors did not state details.	High risk Use of blinding to patients was not applied and blinding of investigators was not reported.	High risk Outcome assessment was based on subjective outcome self-reported by patients themselves.	Low risk All participants completed the study. Drop-out rate: 0%	Unclear risk Protocol is not reported by authors.
Feng 2004	Unclear risk Quote: “65 patients were randomly divided into 2 groups” Random sequence generation method not stated.	Unclear risk Authors did not state details.	High risk Use of blinding to patients was not applied and blinding of investigators was not reported.	High risk Outcome assessment was based on subjective outcome self-reported by patients themselves.	Low risk All participants completed the study. Drop-out rate: 0%	Unclear risk Protocol is not reported by authors.
Wang 2002	High risk Random sequence was generated according to the order of consultation.	Unclear risk Authors did not state details.	High risk Use of blinding to patients was not applied and blinding of investigators was not reported.	High risk Outcome assessment was based on subjective outcome self-reported by patients themselves.	Low risk All participants completed the study. Drop-out rate: 0%	Unclear risk Protocol is not reported by authors.
Wu 2010	High risk Random sequence was generated according to the order of consultation.	Unclear risk Authors did not state details.	High risk Use of blinding to patients was not applied and blinding of investigators was not reported.	High risk Outcome assessment was based on subjective outcome self-reported by patients themselves.	Low risk All participants completed the study. Drop-out rate: 0%	Unclear risk Protocol is not reported by authors.
Sun 2004	Low risk Random sequence was generated from a table of random numbers.	Unclear risk Authors did not state details.	High risk Use of blinding to patients was not applied and blinding of investigators was not reported.	High risk Outcome assessment was based on subjective outcome self-reported by patients themselves.	Low risk All participants completed the study. Drop-out rate: 0%	Unclear risk Protocol is not reported by authors.
Yang 2011	High risk Random sequence was generated according to the order of consultation.	Unclear risk Authors did not state details.	High risk Use of blinding to patients was not applied and blinding of investigators was not reported.	High risk Outcome assessment was based on subjective outcome self-reported by patients themselves.	Low risk All participants completed the study. Drop-out rate: 0%	Unclear risk Protocol is not reported by authors.
Wang 2013	Unclear risk Quote: “80 patients were randomly divided into 2 groups” Random sequence generation method not stated.	Unclear risk Authors did not state details.	High risk Use of blinding to patients was not applied and blinding of investigators was not reported.	High risk Outcome assessment was based on subjective outcome self-reported by patients themselves.	Low risk All participants completed the study. Drop-out rate: 0%	Unclear risk Protocol is not reported by authors.
Zhou 2005	Unclear risk Quote: “126 patients were randomly divided into 2 groups” Random sequence generation method not stated.	Unclear risk Authors did not state details.	High risk Use of blinding to patients was not applied and blinding of investigators was not reported.	High risk Outcome assessment was based on subjective outcome self-reported by patients themselves.	Low risk All participants completed the study. Drop-out rate: 0%	Unclear risk Protocol is not reported by authors.
Sun 2012	Unclear risk Quote: “100 patients were randomly divided into 2 groups” Random sequence generation method not stated.	Unclear risk Authors did not state details.	High risk Use of blinding to patients was not applied and blinding of investigators was not reported.	High risk Outcome assessment was based on subjective outcome self-reported by patients themselves.	Low risk All participants completed the study. Drop-out rate: 0%	Unclear risk Protocol is not reported by authors.
Zheng 2013	Low risk Random sequence was generated from a table of random numbers.	Unclear risk Authors did not state details.	High risk Use of blinding to patients was not applied and blinding of investigators was not reported.	High risk Outcome assessment was based on subjective outcome self-reported by patients themselves.	Low risk All participants completed the study. Drop-out rate: 0%	Unclear risk Protocol is not reported by authors.
Zhou 2013	Unclear risk Quote: “108 patients were randomly divided into 2 groups” Random sequence generation method not stated.	Unclear risk Authors did not state details.	High risk Use of blinding to patients was not applied and blinding of investigators was not reported.	High risk Outcome assessment was based on subjective outcome self-reported by patients themselves.	Low riskAll participants completed the study.Drop-out rate: 0%	Unclear risk Protocol is not reported by authors.
Hu 2012	High risk Random sequence was generated according to the order of consultation.	Unclear risk Authors did not state details.	High risk Use of blinding to patients was not applied and blinding of investigators was not reported.	High risk Outcome assessment was based on subjective outcome self-reported by patients themselves.	Low risk All participants completed the study. Drop-out rate: 0%	Unclear risk Protocol is not reported by authors.
Jin 2013	High risk Random sequence was generated according to the order of consultation.	Unclear risk Authors did not state details.	High risk Use of blinding to patients was not applied and blinding of investigators was not reported.	High risk Outcome assessment was based on subjective outcome self-reported by patients themselves.	Low risk Proportion of drop-out amongst study groups differ by ≤ 10%. 2/72 patients dropped out, 2 in acupuncture group. Drop-out rate: 2.8%	Unclear risk Protocol is not reported by authors.
Chen 2013	Low risk Random sequence was generated from a table of random numbers.	Unclear risk Authors did not state details.	High risk Use of blinding to patients was not applied and blinding of investigators was not reported.	High risk Outcome assessment was based on subjective outcome self-reported by patients themselves.	Low risk All participants completed the study. Drop-out rate: 0%	Unclear risk Protocol is not reported by authors.
Zhang 2009	Low risk Random sequence was generated from a table of random numbers.	Unclear risk Authors did not state details.	High risk Use of blinding to patients was not applied and blinding of investigators was not reported.	High risk Outcome assessment was based on subjective outcome self-reported by patients themselves.	Low risk All participants completed the study. Drop-out rate: 0%	Unclear risk Protocol is not reported by authors.
Yang 2009	Low risk Random sequence was generated from a table of random numbers.	Unclear risk Authors did not state details.	High risk Use of blinding to patients was not applied and blinding of investigators was not reported.	High risk Outcome assessment was based on subjective outcome self-reported by patients themselves.	Low risk All participants completed the study. Drop-out rate: 0%	Unclear risk Protocol is not reported by authors.
Shi 2011	Low risk Random sequence was generated from a table of random numbers.	Unclear risk Authors did not state details.	High risk Use of blinding to patients was not applied and blinding of investigators was not reported.	High risk Outcome assessment was based on subjective outcome self-reported by patients themselves.	Low risk All participants completed the study. Drop-out rate: 0%	Unclear risk Protocol is not reported by authors.
Xu 2014	Unclear risk Quote: “42 patients were randomly divided into 2 groups” Random sequence generation method not stated.	Unclear risk Authors did not state details.	High risk Use of blinding to patients was not applied and blinding of investigators was not reported.	High risk Outcome assessment was based on subjective outcome self-reported by patients themselves.	Low risk All participants completed the study. Drop-out rate: 0%	Unclear risk Protocol is not reported by authors.
He 2012	Unclear risk Quote: “260 patients were randomly divided into 2 groups” Random sequence generation method not stated.	Unclear riskAuthors did not state details.	High risk Use of blinding to patients was not applied and blinding of investigators was not reported.	High risk Outcome assessment was based on subjective outcome self-reported by patients themselves.	Low risk All participants completed the study. Drop-out rate: 0%	Unclear risk Protocol is not reported by authors.
Liu 2011	Low risk Random sequence was generated from a table of random numbers.	Unclear risk Authors did not state details.	High risk Use of blinding to patients was not applied and blinding of investigators was not reported.	High risk Outcome assessment was based on subjective outcome self-reported by patients themselves.	Low risk All participants completed the study. Drop-out rate: 0%	Unclear risk Protocol is not reported by authors.

### Results of pairwise meta-analyses on binary outcome

**Figure 3:**
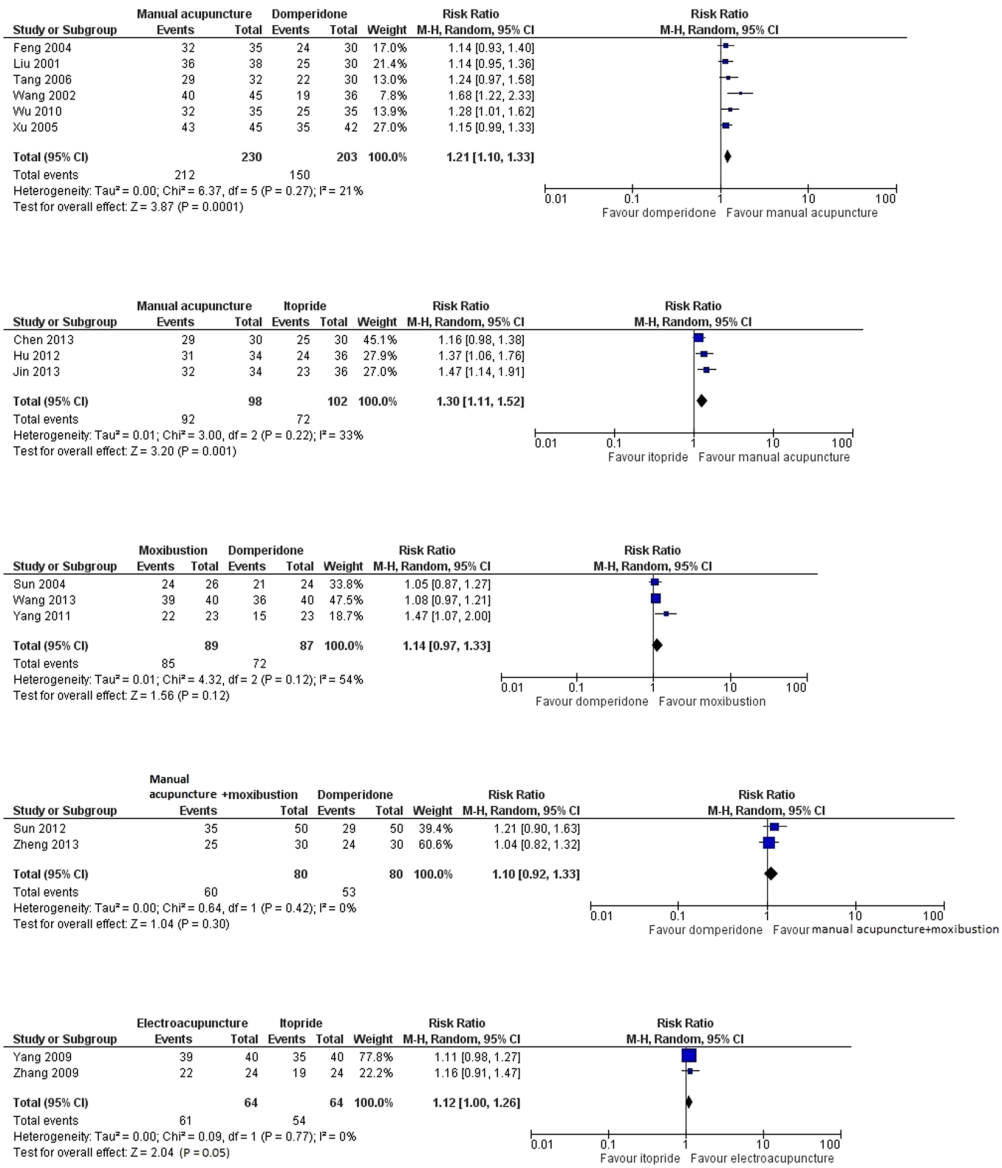
Five pairwise meta-analyses on the comparative effectiveness of acupuncture and related therapies versus prokinetics.

Five pairwise meta-analyses were performed to compare the effectiveness between acupuncture and related therapies versus prokinetics. Detailed results were shown in Fig. 3.

When compared to domperidone, manual acupuncture showed a marginally stronger effect in alleviating global FD symptoms (6 RCTs, pooled RR: 1.21, 95%CI: 1.10, 1.33, p = 0.0001, I^2^ = 21%). Manual acupuncture were also found to be marginally superior to itopride (3 RCTs, pooled RR: 1.30, 95%CI: 1.11, 1.52, p = 0.001, I^2^ = 33%).

There was no statistically significant difference between moxibustion and domperidone in their effectiveness in alleviating global FD symptoms (3 RCTs, pooled RR: 1.14, 95%CI: 0.97, 1.33, p = 0.12, I^2^ = 54%), but moderate level of heterogeneity exists. No significant difference were observed for the following 2 comparisons as well: manual acupuncture plus moxibustion versus domperidone (2 RCTs, pooled RR: 1.10, 95%CI: 0.92, 1.33, p = 0.30, I^2^ = 0%); and electroacupuncture alone versus itopride alone (2 RCTs, pooled RR: 1.12, 95%CI: 1.00, 1.26, p = 0.05, I^2^ = 0%).

### Results of network meta-analysis

**Figure 4:**
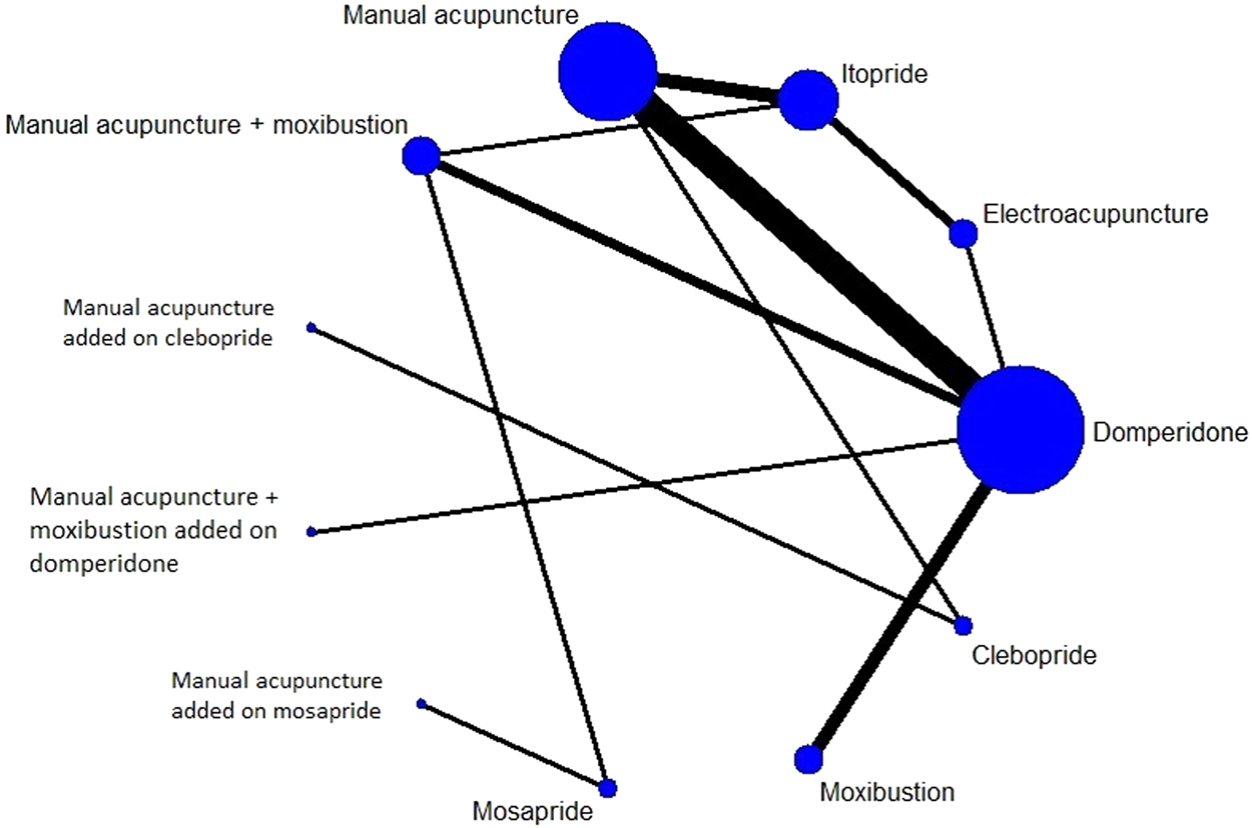
Network of comparison on patient reported global functional dyspepsia symptoms. Width of the lines represents the proportion of the number of trials for each comparison to the number of trials. Size of the nodes represents the proportion of the number of randomized patients (sample sizes).

**Figure 5:**
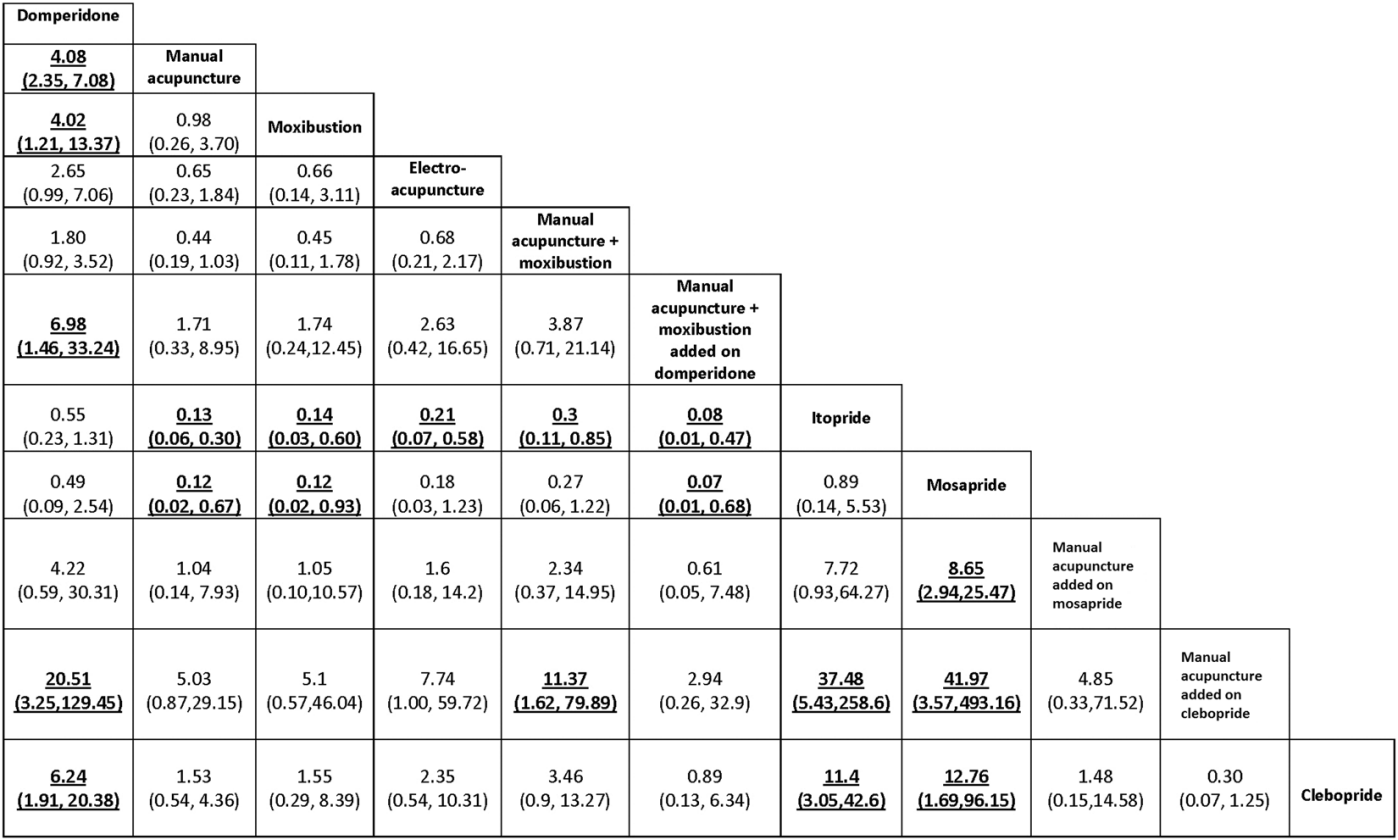
Comparative effectiveness of 11 interventions for alleviating patient reported global functional dyspepsia symptom: Results of indirect comparisons. Results are the relative risks (RRs) and related 95% credibility intervals in the row-defining treatment compared with the RRs in the column-defining treatment. RRs higher than 1 favour the column-defining treatment, and vice versa. Significant result is in bold and underlined.

A network was devised to illustrate the comparative effectiveness among 11 interventions for patient reported global FD symptom. These 11 interventions were different forms of acupuncture and related therapies, used alone or as an add-on to prokinetics. The common comparator among all RCTs in the network was prokinetics (Fig. 4). Indirect comparison on the dichotomous outcome of patient reported global FD symptom alleviation among these 11 treatments is shown in Fig. 5.

Five interventions including (i) manual acupuncture, (ii) moxibustion, (iii) clebopride, (iv) combination of manual acupuncture, moxibustion and domperidone, and (v) combination of manual acupuncture and clebopride, were significantly more effective than domperidone alone (first column of Fig. 5).

Five interventions including (i) manual acupuncture, (ii) moxibustion, (iii) electroacupuncture, (iv) combination of manual acupuncture and moxibustion, and (v) combination of manual acupuncture, moxibustion and domperidone showed superiority over itopride alone (seventh row of Fig. 5).

Four treatments including (i) manual acupuncture, (ii) moxibustion, and (iii) combination of manual acupuncture, moxibustion and domperidone, and (iv) combination of manual acupuncture and mosapride were significantly more effective than mosapride alone (eighth row of Fig. 5, third last row of Fig. 5).

The combination of manual acupuncture and clebopride was significantly more effective than 4 other treatments, including (i) domperidone, (ii) itopride, (iii) mosapride, and (iv) combination of manual acupuncture and moxibustion (second last row of Fig. 5). Lastly, clebopride was more effective than 3 other prokinetics including domperidone, itopride and mosapride (last row of Fig. 5).

**Figure 6:**
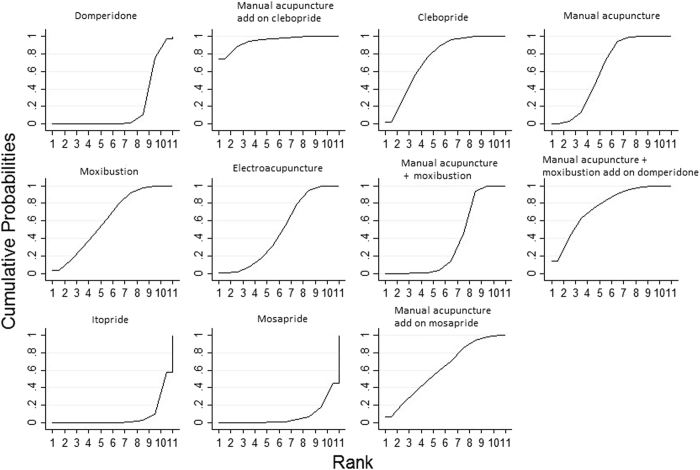
Surface under the cumulative ranking curves (SUCRA) for patient reported global symptom in functional dyspepsia patients. The x-axis represents the possible rank of each treatment (from the first best rank to the worst according to the alleviation of patient reported global functional dyspepsia symptom). The y-axis indicated the cumulative probability for each treatment to be the best treatment, the second best treatment, the third best treatment, and so on.

Figure 6 showed the cumulative probabilities (SUCRA results) of being the best option for alleviating patient reported global FD symptom, when the 11 treatments are compared simultaneously. The combination of manual acupuncture and clebopride has the highest probability being the best (95.0%), followed by the combination of manual acupuncture, moxibustion and domperidone (76.1%), clebopride (74.5%), manual acupuncture (62.6%), moxibustion (62.3%), combination of manual acupuncture and mosapride (61.9%), electroacupuncture (48.6%), combination of manual acupuncture and moxibustion (35.9%), and domperidone (18.4%). Mosapride (7.6%) and itopride (7.1%) have the lowest probabilities.

In this NMA, inconsistency of direct and various indirect effect estimates was insignificant. IF was found to be small (z test for three loops = 0.27, 0.54 and 0.52, all p values > 0.05).

### Results from continuous outcome

We have extracted continuous data on patient reported individual symptoms in continuous data including postprandial fullness, early satiety, epigastric pain and epigastric burning comparing (i) manual acupuncture, (ii) moxibustion, and (iii) combination of manual acupuncture and moxibustion with domperidone. However, due to the scarcity of comparisons in the network, consistency assumption cannot be met and thus we chose not to perform NMA. When compared to domperidone, manual acupuncture showed stronger effect in alleviating postprandial fullness (SMD: −0.79, 95%CI: −1.30, −0.28, p = 0.002)^46^, moxibustion was found to be superior in alleviating both early satiety (SMD: −0.77, 95%CI: −1.37, −0.17, p = 0.01) and epigastric pain (SMD: −0.87, 95%CI: −1.47, −0.26, p = 0.005)^47^, and combination of manual acupuncture and moxibustion was shown to be more favourable in alleviating epigastric pain (SMD: −0.66, 95%CI: −1.18, −0.14, p = 0.01)^48^. However, no statistically significant difference were observed for the following comparisons when compared with domperidone: manual acupuncture in alleviating early satiety (SMD: −0.31, 95%CI: −0.80, 0.18, p = 0.21) and epigastric pain (SMD: −0.13, 95%CI: −0.62, 0.35, p = 0.59)^46^; moxibustion in alleviating postprandial fullness (SMD: −0.11, 95%CI: −0.69, 0.47, p = 0.71) and epigastric burning (SMD: −0.58, 95%CI: −1.17, 0.01, p = 0.06)^47^; and combination of manual acupuncture and moxibustion in alleviating postprandial fullness (SMD: −0.20, 95%CI: −0.71, 0.31, p = 0.43), early satiety (SMD: 0.12, 95%CI: −0.38, 0.63, p = 0.63) and epigastric burning (SMD: −0.21, 95%CI: −0.72, 0.30, p = 0.41)^48^. Detailed results are shown in Appendix 4.

### Adverse events

No serious adverse events were reported in all included RCTs. All reported adverse events were of minor and transient nature. In one included RCT^48^ reported 4 cases of ecchymosis at acupuncture points after manual acupuncture plus moxibustion, while rash (n = 1) and constipation (n = 2) were reported in the domperidone group. In another RCT^49^, there were six patients reporting minor nausea, five cases of increased defecation frequency, and five stomach rumble cases reported as adverse events in the combined manual acupuncture and mosapride group. While in the mosapride group, there were respectively five, four and five cases of minor nausea, increased frequency of defecation and stomach rumble reported as adverse events.

## Discussion

### Implications for practice

From our results, the combination of clebopride plus manual acupuncture and the combination of domperidone plus manual acupuncture and moxibustion are ranked to have the highest probability of being the best treatment options. Taking into account potential adverse effects of these prokinetics, including Parkinsonism syndrome and hemifacial dystonia from clebopride^13, 14^; and extra-pyramidal reactions and cardiac arrhythmic effects from domperidone, manual acupuncture or moxibustion could be alternatives to these medications.

Physiologically, bidirectional brain-gut interactions involve the regulation of digestive processes, including the control of appetite, food in-take, as well as coordination of the gastrointestinal (GI) tract activities^50^. Central and peripheral alterations of brain-gut interactions are proposed to cause symptoms of chronic abdominal pain and associated GI dysfunction^51^. A recent systematic review has shown associations of FD with functional abnormalities in brain-gut interactions, including the aspects of sensory and pain modulation, emotion, saliency and homeostatic processing^52^. A cross-sectional study performed by Zeng and colleagues has compared resting brain activity between FD patients and healthy subjects. Higher glycometabolism was observed among FD patients than healthy subjects in the key regions of the homeostatic afferent processing network, including the insula, hypothalamus, brainstem and the anterior cingulate cortex (ACC). The abnormalities of these regions were significantly related to the severity of FD symptoms^53^.

Increased sensory signal from the gut, as well as impaired central modulation of pain and gut functions were considered to be the key pathogenic features among FD patients. These would subsequently cause central changes like functional brain abnormalities and peripheral changes including visceral hypersensitivity, abnormal gastric motility and accommodation^52^. These mechanisms are found to be associated with FD symptoms^53^.

With regards to the potential therapeutic mechanisms of acupuncture, a RCT comparing brain responses between FD patients receiving acupuncture and sham acupuncture may offer some insights. It is observed that deactivations of glycometabolism in cerebral regions including the insula, ACC, prefrontal cortex, putamen, hypothalamus, hippocampus, parahippocampal gyrus and temporal pole were observed only in patients receiving acupuncture, but not amongst those with sham acupuncture. Since the majority of these deactivated regions in the acupuncture group belonged to the homeostatic sensory processing network, acupuncture is suggested to have an effect in modulating the activity of the homeostatic afferent processing network and hence restoring the balance of homeostatic mechanism. This is a potential mechanism that explain the effectiveness of acupuncture for managing FD^54^.

Since all participants of the included trials were Chinese, applicability of our results to other ethnicity is limited. Also, external validity of our results is limited by the use of heterogeneous diagnostic criteria for inclusion amongst trials. It is noteworthy that the Rome III criteria has been adopted in 11 out of 22 included RCTs, and it is acknowledged that its use may lead to exclusion of a substantial number of patients with endoscopically verified FD^55^. The application of such a strict inclusion criteria implies that trial patients are likely to differ from average patient seen in clinical practice^55^. In the newly announced Rome IV criteria, only minor modifications were made with regards to symptom description^56^. In the future, a more flexible diagnostic criteria might be used in recruiting patients in FD clinical trials^57^.

### Implications for research

With regards to internal validity, our assessment suggested that risk of bias among included RCTs is often unclear due to poor reporting, especially in allocation concealment and selective outcome reporting domains. Indeed, poor reporting is a prevalent problem in Chinese medicine publications^58^ and we cannot draw any solid conclusion on their methodological rigor. Also, end points are subjective patient reported outcomes, but blinding of patients and investigators were not applied among Included RCTs. These risks of bias may lead to an exaggeration of treatment effects for acupuncture and related therapies^59^.

On top of improving rigor, future trialists should adhere to the CONSORT reporting statement^60^ for improving the usefulness of study results, as well as in methodological transparency. All adverse events should also be well reported. With regards to outcome selection, while patient reported symptoms alleviation can remain as one of the endpoint, depending on such dichotomous assessment is not sufficient. Both objective as well as detailed patient centered outcome should be reported in future trials, including (i) individual symptom assessment; (ii) disease specific quality of life questionnaire; (iii) nutrient drink test; and (iv) gastric emptying test^61^. Also, follow-up duration of 16 out of 22 included RCTs was only 4 weeks. Longer term benefits of acupuncture and related therapies should be evaluated by following the recommended follow-up duration of at least 12 weeks^61^.

Current guideline recommends PPIs as one of the first line treatment for FD^62^, but in this systematic review we did not locate any RCTs which provide evidence on the comparative effectiveness of acupuncture and related therapies and PPIs, or their combined use. Acupuncture and related therapies could also be an alternative to patient responding poorly to PPIs, or when patients are contraindicated for its adverse effects of increased risk of dementia^63^, chronic and acute kidney disease, hypomagnesemia, *Clostridium difficile* infection, and osteoporotic fractures^64^. Future trials should investigate this research question.

Our assessment indicated rooms for improvement on rigor of included SRs. There are several methodological areas that require attention from future SR authors. These include assessment of publication bias, reporting of conflict of interest, searching for unpublished studies, providing a list of included and excluded studies, and publishing protocols of SRs. Methodological and reporting standards of SR should follow the AMSTAR tool and PRISMA statement^65^ respectively. Finally, although mechanism of acupuncture’s effect on FD has been studied, therapeutic mechanism of moxibustion has not been studied and is subjected to further research.

## Conclusion

With clinical evidence summarized by this overview of SRs and NMA, it is observed that the combination of manual acupuncture and clebopride has the highest probability of being the most effective therapy for alleviating FD symptoms. FD patients who are intolerant or unresponsive to prokinetics, manual acupuncture or moxibustion may be used as alternative. The potential synergistic effect of PPIs plus acupuncture and related therapies should also be explored in future trials. Future trialists should pay attention to choice of diagnostic criteria, outcome assessment, as well as methodological rigor in trial design.

## Electronic supplementary material

Appendix 1–4
