# Supplementary material for: Acupuncture and related therapies used as add-on or alternative to prokinetics for functional dyspepsia: overview of systematic reviews and network meta-analysis
Source: Sci Rep. 2017 Sep 4;7:10320. doi: 10.1038/s41598-017-09856-0 (PMC5583250; doi:10.1038/s41598-017-09856-0)
Supplement: Supplementary file 1 — Appendix 1–4 [file 41598_2017_9856_MOESM1_ESM.pdf]

Acupuncture and related therapies used as add-on or alternative to prokinetics for functional dyspepsia: overview of systematic reviews and network meta-analysis

Robin ST Ho [a], Vincent CH Chung [a, b,c], Charlene HL Wong [b], Justin CY Wu [b], Samuel YS Wong [a], Irene XY Wu [b,c]

a. Jockey Club School of Public Health and Primary Care, The Chinese University of Hong Kong, Hong Kong

b. Hong Kong Institute of Integrative Medicine, The Chinese University of Hong Kong, Hong Kong

c. Cochrane Hong Kong, The Chinese University of Hong Kong

Corresponding author: Vincent CH Chung (vchung@cuhk.edu.hk)

Appendix 1: Search strategies and results for systematic reviews on acupuncture for functional dyspepsia

i) Cochrane Database of Systematic Reviews (CDSR) from inception to 2/11/2015

1	dyspepsia*.mp.	211
2	functional dyspepsia*.mp.	12
3	1 or 2	211
4	acupunctur*.mp.	441
5	electroacupunctur*.mp.	85
6	electro-acupunctur*.mp.	57
7	acupoint*.mp.	66
8	Transcutaneous Electric Nerve Stimulat*.mp.	40
9	percutaneous electrical nerve stimulat*.mp.	4
10	TENS.mp.	151
11	4 or 5 or 6 or 7 or 8 or 9 or 10	516
**12**	**3 and 11**	**9**

ii) Database of Abstracts of Reviews of Effects (DARE) from inception to 2/11/2015

1	dyspepsia*.mp.	113
2	functional dyspepsia*.mp.	34
3	1 or 2	113
4	acupunctur*.mp.	471
5	electroacupunctur*.mp.	56
6	electro-acupunctur*.mp.	26
7	Transcutaneous Electric Nerve Stimulat*.mp.	52
8	percutaneous electrical nerve stimulat*.mp.	1
9	TENS.mp.	58
10	4 or 5 or 6 or 7 or 8 or 9	531
**11**	**3 and 10**	**3**

iii) MEDLINE from inception to 2/11/2015

1	search:.tw.	247174
2	meta analysis.mp,pt.	88048
3	review.pt.	2021893
4	di.xs.	4776296
5	associated.tw.	2397503
6	1 or 2 or 3 or 4 or 5	7851644
7	exp Dyspepsia/	7601
8	dyspepsia*.mp.	11191
9	functional dyspepsia*.mp.	2039
10	7 or 8 or 9	11191
11	exp Acupuncture/	1355
12	acupunctur*.mp.	19909
13	exp Acupuncture Points/	4554
14	exp Acupuncture Therapy/	18486
15	exp Acupuncture Analgesia/	1104
16	exp Electroacupuncture/	2846
17	electroacupunctur*.mp.	3494
18	electro-acupunctur*.mp.	609
19	acupoint*.mp.	2913
20	exp Transcutaneous Electric Nerve Stimulation/	6398
21	Transcutaneous Electric Nerve Stimulat*.mp.	3659
22	percutaneous electrical nerve stimulat*.mp.	38
23	TENS.mp.	7862
24	11 or 12 or 13 or 14 or 15 or 16 or 17 or 18 or 19 or 20 or 21 or 22 or 23	31271
25	6 and 10 and 24	25
26	Limit 25 to humans	25

iv) EMABSE from inception to 2/11/2015

1	exp methodology/	4137348
2	search:.tw.	350453
3	review.pt.	2110277
4	1 or 2 or 3	6045393
5	exp dyspepsia/	27468
6	dyspepsia*.mp.	30108
7	functional dyspepsia*.mp.	3819
8	5 or 6 or 7	30108
9	exp acupuncture/	36743
10	acupunctur*.mp.	36396
11	exp acupuncture analgesia/	1413
12	exp acupuncture needle/	437
13	exp electroacupuncture/	4665
14	electroacupunctur*.mp.	5436
15	electro-acupunctur*.mp.	980
16	acupoint*.mp.	4391
17	exp transcutaneous nerve stimulation/	6216
18	Transcutaneous Electric Nerve Stimulat*.mp.	302
19	percutaneous electrical nerve stimulat*.mp.	68
20	TENS.mp.	11033
21	9 or 10 or 11 or 12 or 13 or 14 or 15 or 16 or 17 or 18 or 19 or 20	52935
22	4 and 8 and 21	152
**23**	**limit 22 to human**	**150**

v) Wan Fang Digital Journals [Chinese] from inception to 2/11/2015

("系统综述" OR "荟萃分析" OR "META") AND ("针灸" OR "针刺" OR "电针" OR "耳针" OR “头针” OR “水针”) AND ("功能性消化不良" OR "消化不良")

(Yielded 3 citations)

vi) China National Knowledge Infrastructure [Chinese] from inception to 2/11/2015

(KY='系统综述' OR KY='荟萃分析' OR KY='META') AND (KY='针灸' OR KY='针刺' OR KY='电针' OR KY='耳针' OR KY='头针' OR KY='水针') AND (KY='功能性消化不良' OR KY='消化不良')

(Yielded 2 citations)

vii) Taiwan Periodical Literature Databases [Chinese] from inception to 2/11/2015

(TX=系統綜述 OR 薈萃分析 OR META) [AND] (TX=針灸 OR 針刺 OR 電針 OR 耳針 OR 頭針 OR 水針) [AND] (TX=功能性消化不良 OR 消化不良)

(Yielded 0 citations)

viii) Chinese Biomedical Database (CBM) [Chinese] from inception to 2/11/2015

("系统综述"[全字段] OR "荟萃分析"[全字段] OR "META"[全字段]) AND ("针灸"[全字段] OR "针刺"[全字段] OR "电针"[全字段] OR "耳针"[全字段] OR “头针”[全字段] OR “水针”[全字段]) AND ("功能性消化不良"[全字段] OR "消化不良"[全字段])

(Yielded 0 citations)

Appendix 2: Lists of included systematic reviews

1. Lan L, Zeng F, Liu GJ, Ying L, Wu X, Liu M, et al. Acupuncture for functional dyspepsia. Cochrane Database Syst Rev. 2014;10:CD008487.

2. Kim KN, Chung SY, Cho SH. Efficacy of acupuncture treatment for functional dyspepsia: A systematic review and meta-analysis. Complementary Therapies in Medicine. 2015;23(6):759-66.

3. Zhou W, Su J, Zhang H. Efficacy and Safety of Acupuncture for the Treatment of Functional Dyspepsia: Meta-Analysis. The Journal of Alternative and Complementary Medicine. 2016;22(5):380-89.

4. Wu XW, JI HZ, XU LE, Wang FY. The effect of acupuncture and moxibustion on functional dyspepsia compared with prokinetic agents: a meta-analysis. Chin J Integr Trad West Med Dig. 2015; 23(2):100-04.

5. Zhu H. Evaluation of acupuncture on functional dyspepsia: a systematic review. Chengdu University of Traditional Chinese Medicine (Master thesis). 2008 April. (Citation translated from Chinese to English)

Appendix 3: Lists of included randomized controlled trials

1. Tang SX, Xu ZH, Tang P. A comparative study of functional dyspepsia treated with acupuncture. Journal of Sichuan of Traditional Chinese Medicine. 2006;24(4):101-02. (Citation translated from Chinese to English).

2. Liu WQ, Wang J, Hao ZY. Clinical study on effect of acupuncture on gastrointestinal motility in the patient of functional dyspepsia. Chinese Acupuncture and Moxibustion. 2001 May;21(5):267-69.

3. Xu GX, Liu YB. Clinical study on functional dyspepsia treated with acupuncture. Modern Journal of Integrated Traditional Chinese and Western Medicine. 2005 December;14(23):3076-77. (Citation translated from Chinese to English).

4. Feng GX, Zhu Y, Jiang GF. Observation on therapeutic effect of 35 cases of functional dyspepsia treated by acupuncture. New Journal of Traditional Chinese Medicine. 2004;36(1):48-49. (Citation translated from Chinese to English).

5. Wang ZC, Wang WL. Observation on therapeutic effect of elongated needle therapy on non-ulcerative dyspepsia. Chinese Acupuncture and Moxibustion. 2002;22(3):149-50. (Citation translated from Chinese to English).

6. Wu L. Observation on therapeutic effect of Shugan Tiao - pi spleen acupuncture in treating functional dyspepsia. Jilin Journal of Traditional Chinese Medicine. 2010;30(10):885-886. (Citation translated from Chinese to English).

7. Sun SB, Yang J. Observation on the therapeutic effect of bird-pecking moxibustion of specific acupoints in the treatment of functional dyspepsia. World Journal of Acupuncture & Moxibustion.2004;14(2):15-20.

8. Yang JM, Zhang TF, Huang GF. Treatment 23 cases functional dyspepsia by moxibustion treatment. Journal of Jiangxi Chinese Medicine. 2011;42(1):43-45. (Citation translated from Chinese to English).

9. Wang YJ, Wang LJ. Matrix moxibustion in the treatment for 40 cases of functional dyspepsia of spleen-stomach qi deficiency pattern. Western Journal of Tradidtional Chinese Medicine. 2013;26(3):60-61

10. Zhou Y, Zheng JG. Clinical observation on acupuncture treatment of functional dyspepsia. Journal of Acupuncture and Tuina Science. 2005;3(2):20-22.

11. Sun JR. Clinical observation on 50 cases of functional dyspepsia treated by warming acupuncture and moxibustion. Chinese Journal of ethnomedicine and ethnopharmacy. 2012;21(7):95-96.

12. Zheng X, Song X, Jiang X, Zhang GZ, Chen JQ. Clinical observation of acupuncture for treatment of Pi Wei Qi deficiency functional dyspepsia. J Trop Med. 2013;13:232-34.

13. Zhou KR. Clinical observation on 108 cases of functional dyspepsia treated by acupuncture and moxibustion. China Health Care & Nutrition. 2013;23(2):935. (Citation translated from Chinese to English).

14. Hu Y. Therapeutic effect observation on functional dyspepsia treated with acupuncture by differentiation of symptoms and signs and its effect on serum gastrin. Hubei University of Chinese Medicine (Master thesis). 2012 May. (Citation translated from Chinese to English)

15. Jin L, Gao ZC, Zhou L, Zhang WC, Zhang HX. Clinical curative effect evaluation of acupuncture by syndrome differentation for functional dyspepsia. Liaoning Journal of Traditional Chinese Medicine. 2013;40(6):1222-25. (Citation translated from Chinese to English).

16. Chen QP. Clinical study on acupuncture for smoothing the liver and regulating the stomach in the treatment of functional dyspepsia. Hubei University of Chinese Medicine (Master thesis). 2013 May. (Citation translated from Chinese to English).

17. Zhang W. Clinical observation of Shu Mu with electroacupuncture treatment on functional dyspepsia. Hubei University of Chinese Medicine (Master thesis). 2009 May. (Citation translated from Chinese to English)

18. Yang M. Electroacupuncture treatment of functional dyspepsia from clinical research. Hubei University of Chinese Medicine (Master thesis). 2009 May. (Citation translated from Chinese to English).

19. Shi L. Clinical study of the combination of acupuncture and moxibustion at heat sensitive points on functional dyspepsia. Hubei University of Chinese Medicine (Master thesis). 2011 May. (Citation translated from Chinese to English).

20. Xu BL, Wu W, & Zhang WQ. Observations on the therapeutic effect of warming acupuncture on functional dyspepsia with spleen Stomach qi deficiency. Zhejiang Journal of Traditional Chinese Medicine. 2014 January;49(1):58. (Citation translated from Chinese to English).

21. He CL. Clinical efficacy of mosapride and acupuncture used in the treatment of FD. Journal of Qiqihar University of Medicine. 2012;33(21):2906-07.

22. Liu CH, Shu J. Clinical curative effect observing of acupuncture combined with clebopride on functional dyspepsia. Medical Innovation of China. 2011;8(22):3-4. (Citation translated from Chinese to English).

Appendix 4: Comparative effectiveness of acupuncture and related therapies versus prokinetics for alleviating patient reported individual functional dyspepsia symptoms.

Outcome measurement	First author, year of publication (Country)	Interventions	Mean ± SD after intervention #	Controls	Mean ± SD after control intervention#	Standardized mean differences between groups (95%CI)	P values* of the standardized mean difference
**Postprandial fullness**	Feng 2004 (China)	Manual acupuncture	0.93 ± 0.79	Domperidone	1.55 ± 0.76	-0.79 (-1.30, -0.28)	0.002^
	Yang 2011 (China)	Moxibustion	0.83 ± 0.49	Domperidone	0.88 ± 0.39	-0.11 (-0.69, 0.47)	0.71
	Zheng 2013 (China)	Manual acupuncture + moxibustion	0.63 ± 0.93	Domperidone	0.83 ± 1.02	-0.20 (-0.71, 0.31)	0.43
**Early satiety**	Feng 2004 (China)	Manual acupuncture	1.29 ± 0.62	Domperidone	1.47 ± 0.51	-0.31 (-0.80, 0.18)	0.21
	Yang 2011 (China)	Moxibustion	0.73 ± 0.34	Domperidone	1.05 ± 0.47	-0.77 (-1.37, -0.17)	0.01^
	Zheng 2013 (China)	Manual acupuncture + moxibustion	1.00 ± 1.02	Domperidone	0.87 ± 1.04	0.12 (-0.38, 0.63)	0.63
**Epigastric pain**	Feng 2004 (China)	Manual acupuncture	0.81 ± 0.52	Domperidone	0.89 ± 0.66	-0.13 (-0.62, 0.35)	0.59
	Yang 2011 (China)	Moxibustion	0.68 ± 0.38	Domperidone	1.02 ± 0.39	-0.87 (-1.47, -0.26)	0.005^
	Zheng 2013 (China)	Manual acupuncture + moxibustion	0.50 ± 0.68	Domperidone	0.97 ± 0.72	-0.66 (-1.18, -0.14)	0.01^
**Epigastric burning**	Yang 2011 (China)	Moxibustion	0.41 ± 0.47	Domperidone	0.68 ± 0.45	-0.58 (-1.17, 0.01)	0.06
	Zheng 2013 (China)	Manual acupuncture + moxibustion	0.13 ± 0.43	Domperidone	0.23 ± 0.50	-0.21 (-0.72, 0.30)	0.41

Post interventions value with symptom severity assessed based on a range of score from 0 to 3 (0 represents the least severity, 1 represents low severity, 2 represents moderate severity and 3 represents high severity) ; * Statistically significant with a 2-tailed significance level of 0.05; ^ Statistically significant standardized mean difference.

Keys: SD: standard deviation, CI: Confidence interval.
